# Mathematical modeling analysis on the dynamics of university students animosity towards mathematics with optimal control theory

**DOI:** 10.1038/s41598-022-15376-3

**Published:** 2022-07-08

**Authors:** Shewafera Wondimagegnhu Teklu, Birhanu Baye Terefe

**Affiliations:** Department of Mathematics, Collage of Natural and Computational Sciences, Debre Berhan University, Debre Berhan, Ethiopia

**Keywords:** Computational biology and bioinformatics, Mathematics and computing

## Abstract

Animosity towards mathematics is a very common worldwide problem and it is usually caused by wrong information, low participation, low challenge tolerance, falling further behind, being unemployed, and avoiding the advanced math classes needed for success in many careers. In this study, we have considered and formulated the new SEATS compartmental mathematical model with optimal control theory to analyze the dynamics of university students’ animosity towards mathematics. We applied the next-generation matrix, Ruth-Hurwitz criteria, Lyapunov function, and Volterra-Lyapunov stable matrices to show local and global stability of equilibrium points of the model respectively. The study demonstrated that the animosity-free equilibrium point is both locally and globally asymptotically stable whenever the model basic reproduction number is less than unity, whereas the animosity-dominance equilibrium point is both locally and globally asymptotically stable when the model basic reproduction number is greater than unity. Finally, we applied numerical ode45 solvers using the Runge–Kutta method and we have carried out numerical simulations and shown that applying both prevention and treatment controls is the best strategy to minimize and possibly eradicate the animosity-infection in the community under consideration.

## Introduction

Mathematics is everywhere in individuals life and culture throughout the world^28^. Students’ attitudes typically defined as consisting of cognitive (beliefs), affective (emotions), and conative (behavior) dimensions and play a key role in their academic accomplishment and their mental attitude may maximize their capability in the subject matter^1,11^. In other words attitudes toward mathematics have been defined as lovingness or disliking of mathematics, a trend to engage in or quash mathematical activities, a belief that one is beneficial or not beneficial at mathematics, and a belief that mathematics is important or useless and it can be classified into four various appraising terms: the emotions that the student automatically associates with the concept 'mathematics, evaluations of situations that the student expects to follow as a result of performing mathematics, the emotions that the student goes through during mathematics-related actions, and the value of mathematics-related goals in the students’ global aim structure^8–10^. The students’ attitudes towards mathematics impact their academic accomplishment; thus, a more mental attitude may maximize their capability in the subject matter^1^. Preventions and control measures against animosity such as cognitive tutors, intelligent tutoring systems, psychological treatment, and adaptive learning environments are all variations of the same common theme: instructional systems that hold empiric models of the student to estimate student conducts and knowledge and to act upon these estimates to make a pedagogic movement as students’ bring forward towards earning expertise and domination of the target area^3^.

Mathematical knowledge and skills are crucial for the scientific and technological development and economic success of societies, for world countries. This is because mathematics skills are very widely essential in understanding other disciplines including social sciences, engineering, sciences, arts, and outspread to all areas of science, technology as well as business enterprises and hence mathematics has been became a key in all sciences^27^. Poor mathematical skills in students depressed them from a large number of professions because mathematical background knowledge is the pre requisite for entrance in any profession^12,22^. Those with low mathematics abilities are likely to have a more negative attitude towards mathematics and will not have the tendency to amend their mathematics skill. Researches indicated that poor attitudes towards mathematics are related to lower levels of achievement in the subject^26^. The main cause of the animosity towards mathematics especially in developing nations are attitudes of the learners, uninteresting lessons and low motivation of teachers attitude, confidence of learners, lack of teaching experiences, economic conditions, parents' educational level, teacher competency in math education, teacher’s personality, intellectual factor, communication, stress, the pressure to perform well, lack of appropriate teaching methods, many numbers of student in the class over demanding tasks^21,27^. Even though, decreasing numbers of students choosing to study mathematics and natural science have been major issue, mathematics has been as a fundamental forerunner to success in worldwide modern society^5,21^. Since 1995, many theoretical researches have been conducted a series of international assessments of educational achievement in mathematics and science^31^.

Mathematical modeling approach using deterministic method^7,13,16,19,23,30^ or stochastic method^11,18,32–34^ or fractional order method^20,24,25^ have been scientific efforts to link and discover real world situations using mathematical models which have fundamental decision-making tools for the analysis of dynamics of communicable diseases and can be used for analyzing a number of real-world physical dynamical situations. The main purpose of this study is going to develop and analyze the new compartmental mathematical model with optimal control theory to analyze the animosity of higher institutions especially university students towards mathematics with tutorial and psychological treatment prevention and control measures. This mathematical model study is a first attempt in the thematic area, to carry out the proposed study we have faced a lack of mathematical modeling analysis literatures on higher institutions (university) students’ animosity against mathematics and hence we have reviewed other literatures that are nearly related in our study. Mamo, Dejen Ketema, 2020^19^ constructed a new Susceptible, Exposed, Infected Deny (SEID) racism expansion compartmental mathematical model, that explains the racism dissemination throughout a community under consideration. His study was mainly concentrated on the racism transmission on societal networks. He has been constructed and examined his proposed model theoretically and verified numerically. Applying the next-generation matrix approach, he has got the basic reproduction number of the model and the model basic reproduction number is almost correlated to the expansion of racism. Kooken, Janice W, et al. 20121^15^ developed and presented the results of growth and establishment of the Cyclical Self-Regulated Learning (SRL) model simulation, a model of student knowledge and metacognitive learning mathematics experiences within an intelligent tutoring system (ITS). Their analysis results provide the establishment of the Cyclical SRL Model, emotion, performance in the ITS, and confirming the interplay of grit. Their simulation model enables mathematical simulations depicting a variety of student scope types and prevention styles and confirming deeper future student learning explorations. Van der Merwe, A, et al. 2018^35^ presented interconnected algorithmic results that utilize mathematical programming models to bring forth and provide learning feedback in the form of academic performance status reports. Yadav, Anuradha, Prashant K. Srivastava, and Anuj Kumar^36^ proposed and analyzed (PSQ) model where P stands potential smokers, S stands smokers and Q stands quitters for understanding of the dynamics of smoking behavior in a population. They first assumed that smokers are quitting smoking which is influenced by the level of determination of individuals. Higher degree of determination will lead to less chances of relapsing. Further the impact of education on potential smokers is also considered. They have shown that when individuals are educated about the fatality of the diseases caused by smoking they will refrain from smoking in future. Khyar, Omar, Jaouad Danane, and Karam Allali^14^ explored mathematically the dynamics of giving up smoking behavior. For this purpose, they performed a mathematical analysis of a smoking model and suggested some conditions to control this serious burden on public health. The model under consideration describes the interaction between the potential smokers $(P),$ the occasional smokers $(L),$ the chain smokers $(S),$ the temporarily quit smokers (${Q}_{T}$), and the permanently quit smokers (${Q}_{P}$). Existence, positivity, and boundedness of the proposed problem solutions are proved. Local stability of the equilibriums is established by using Routh–Hurwitz conditions. Moreover, the global stability of the same equilibriums is fulfilled through using suitable Lyapunov functional. In order to study the optimal control of their problem, they took into account a two controls’ strategies, the government prohibition of smoking in public areas which reduces the contact between nonsmokers and smokers, the educational campaigns and the increase of cigarette cost which prevents occasional smokers from becoming chain smokers. The existence of the optimal control pair is discussed, and by using Pontryagin’s minimum principle, these two optimal controls are characterized. Finally, numerical simulations are performed in order to check the equilibriums stability, confirm the theoretical findings, and show the role of optimal strategy in controlling the smoking severity. Alkhudhari, Zainab, Sarah Al-Sheikh, and Salma Al-Tuwairqi^2^ derived and analyzed a mathematical model of smoking in which the population is divided into four classes: potential smokers, smokers, temporary quitters, and permanent quitters. In their model they studied the effect of smokers on temporary quitters. Two equilibriums of the model are found: one of them is the smoking-free equilibrium and the other corresponds to the presence of smoking. They examined the local and global stability of both equilibriums and support their results by using numerical simulations.

The main limitations of this study are: using non-modelling studies scholars shown us the problem is common in the community, however, in this study we faced problems to find more related literatures to the study and well organized real data of university students’ animosity towards mathematics. Studies of researchers we reviewed did not considered and developed university students’ animosity against mathematics mathematical modelling analysis and hence it makes this study has never been done by other scholars abroad. In our study we formulated and analyzed a new compartmental mathematical model with optimal control theory to minimize and possibly eradicate university students’ animosity towards mathematics from the community and which shows the novelty of the study in the thematic area. Therefore, we are motivated by the limitations of the study to undertake this study and to fulfill the gap. The remaining part of the study is organized as; the deterministic model is constructed in Sect. Deterministic model formulation and is analyzed in Sect. Qualitative investigation of the deterministic model; the stochastic touch is explained in Sect. The transition probability; optimal control analysis is carried out in Sect. Optimal control analysis of the deterministic model; numerical analysis has been performed in Sect. Numerical analysis, and conclusion of the study are carried out in Sect. Conclusion respectively.

## Deterministic model formulation

In this study we considered the total number of students $N(t)$ in a given time $t$ and we divide it in to four disjoint classes. Those are susceptible, exposed, animosity infected and treated classes denoted by S, E, A, and T respectively. The state variables are describe as follows.

i. Animosity-susceptible students. Those are a group of student who are entering in to the university without a strong feeling of dislike or hatred of mathematics includes students who are interested in mathematics and became animosity infected whenever they meet with infected groups and it is denoted by $S(t)$.
ii. Animosity-exposed students. Those are a group of student who are taking mathematics course in the university and have closed contact with animosity student and hearing bad attitude towards mathematics and it is denoted by $E(t).$ Those animosity exposed individuals may be animosity-infected or not.
iii. Animosity-infected students. Those are a group of students who have a strong feeling of dislike or hatred or enmity that tends to display itself in mathematics and score lower grade in the university and it is denoted by $A(t).$
iv. Animosity-treated students. Those are a group of students who are taking different tutorial and psychological treatments by their advisor and lecturers, we call it treated and denoted by $T(t).$

Basic assumptions and parameters definitions of the model

- The animosity-susceptible group $S\left(t\right)$ increases by student who are newly entering in to the university with the rate $\Lambda$, and by the number of student who are purely accept psychological treatment of their mentor and take their tutorial effectively with the rate of $\alpha$, decreases due to the contact of animosity infected students with the rate $\beta$ where the total number of students is constant with equal birth and death rate $\mu$.
- The animosity-exposed group E(t) increases by the students who have closed contact with animosity infected students and hearing about the animosity of mathematics with the rate $\beta$ and decreases by the rate $\gamma$ who are animosted mathematics.
- The animosity infected group A(t) is increases by the rate $\gamma$ and decreases by the tutorial and psychological treatment rate $\delta$ at any time t.
- The treated group T(t) increases by the psychological treatment rate $\delta$ and decreases by the conversion rate $\alpha$ from treated class at any time t.
- Students in all compartments are decrease by the natural death rate $\mu$. Assume the total number of students is constant.
- The parameter $\Lambda =\mu N$ is the recruitment rate of freshman university students.

**Figure 1:**
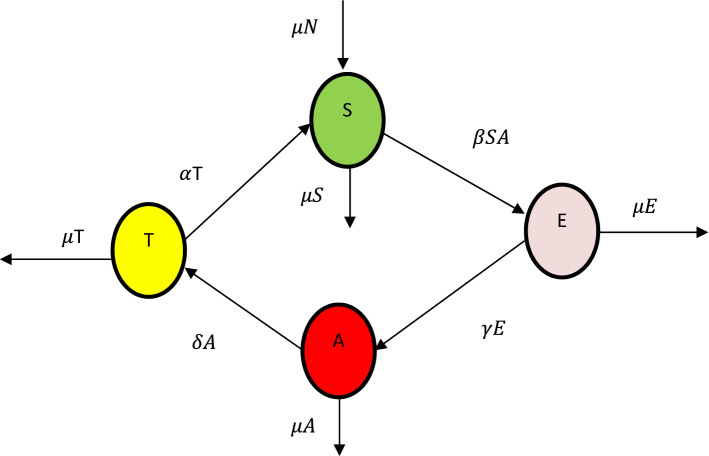
Schematic diagram for flow of the students’ dynamics.

Based on the model assumptions and descriptions above the flow chart (schematic diagram) of the flow of students is given in Fig. 1.

Using the model assumptions, parameter definitions and Fig. 1 the dynamical system of the study is given by

1 $$\begin{gathered} \frac{dS}{{dt}} = {\Lambda } + \alpha T - \beta SA - \mu S, \hfill \\ \frac{dE}{{dt}} = \beta SA - \left( {\mu + \gamma } \right)E , \hfill \\ \frac{dA}{{dt}} = \gamma E - \left( {\mu + \delta } \right)A, \hfill \\ \frac{dT}{{dt}} = \delta A - \left( {\mu + \alpha } \right)T. \hfill \\ \end{gathered}$$

We considered all model parameter values are non-negative and to analyze the mathematical model simply, the variables of the model (1) can be scaled as

2 $$s\left( t \right) = \frac{{{\text{S}}\left( t \right)}}{{{\text{N}}\left( t \right)}} , e\left( t \right) = \frac{E\left( t \right)}{{{\text{N}}\left( t \right)}},v\left( t \right) = \frac{A\left( t \right)}{{{\text{N}}\left( t \right)}},z\left( t \right) = \frac{{{\text{T}}\left( t \right)}}{{{\text{N}}\left( t \right)}},$$

After substituting the time derivative of system (2) in system (1), we have got the new simplified system given by

3 $$\begin{gathered} \frac{ds}{{dt}} = \mu + \alpha z - \beta sv - \mu s, \hfill \\ \frac{de}{{dt}} = \beta sv - \left( {\mu + \gamma } \right)e, \hfill \\ \frac{dv}{{dt}} = \gamma e - \left( {\mu + \delta } \right)v , \hfill \\ \frac{dz}{{dt}} = \delta v - \left( {\mu + \alpha } \right)z, \hfill \\ \end{gathered}$$

The total number of university students is constant, i.e.

$${\text{N}}\left( t \right) = {\text{S}}\left( t \right) + E\left( t \right) + A\left( t \right) + {\text{T}}\left( t \right) = N.$$

Due to the scaled model of system (1), we have got the total number of students as

$$s\left( t \right) + e\left( t \right) + v\left( t \right) + z\left( t \right) = 1.$$

Then the mathematically and biologically feasible domain of the system (3) is

$${\Phi } = \left\{ {\left( {s, e, v, z} \right):s + e + v + z = 1} \right\}.$$

The model is both mathematically and biologically meaningful if following important lemma holds.

### Lemma 1

The set $\Phi$ is positively invariant to the system (3).

### *Proof*

Symbolize $y\left(t\right)={\left(s\left(t\right), e\left(t\right), v\left(t\right), z\left(t\right)\right)}^{T}$ and the system (3) can be rewritten as.

$$\frac{dy\left( t \right)}{{dx}} = f\left( {y\left( t \right)} \right),$$

where $$f(y\left(t\right))={[\left(\mu +\alpha z-\beta sv-\mu s, \beta sv-\left(\mu +\gamma \right)e, \gamma e-\left(\mu +\delta \right)v, \delta v-\left(\mu +\alpha \right)z\right)]}^{T}.$$

Obviously the feasible region $\Phi$ is closed set, and we want to prove if the initial condition $f\left(0\right)\in\Phi$, then the solution $f\left(t\right)\in\Phi$ for all $t\ge 0$.

Take $\partial\Phi$ consists five the hyperspaces ${P}_{1}$,${P}_{2}, {P}_{3}{, P}_{4}$, ${P}_{5}$ such that

$$P_{1} = \left\{ {\left( {s,e, v, 0} \right):s,e, v \in \left[ {0, 1} \right],s + e + v \le 1} \right\},$$

$$P_{2} = \left\{ {\left( {s, e, 0, z} \right):s,e, z \in \left[ {0, 1} \right],s + e + z \le 1} \right\},$$

$$P_{3} = \left\{ {\left( {s, 0, v,z} \right):s, v, z \in \left[ {0, 1} \right],s + v + z \le 1} \right\},$$

$$P_{4} = \left\{ {\left( {0,e, v,z} \right): e, v, z \in \left[ {0, 1} \right], e + v + z \le 1} \right\},$$

$$P_{5} = \left\{ {\left( {s,e, v,z} \right): s, e, v, z \in \left[ {0, 1} \right],s + e + v + z \le 1} \right\},$$

With outer normal vectors ${w}_{1}=\left(0, 0, 0, -1\right)$; ${w}_{2}=\left(0, 0, -1, 0\right)$; ${w}_{3}=\left(0,-1, \mathrm{0,0}\right)$; ${w}_{4}=\left(-\mathrm{1,0}, 0, 0\right)$; ${w}_{5}=\left(\mathrm{1,1}, 1, 1\right)$ respectivelly.

If the dot product of $f(y)$ the and normal vectors (${w}_{1}$;${w}_{2}$;${w}_{3}$;${w}_{4};{w}_{5}$) of the boundary lines are less than zero then $y\left(t\right)\in\Phi$ for all $t\ge 0$.i.e.;

$$f\left( {y\left( t \right)} \right)| _{{y\left( t \right) \in P_{1} }} ,w_{1} = - \delta v + \left( {\mu + \alpha } \right)z \le 0$$

$$f\left( {y\left( t \right)} \right)| _{{y\left( t \right) \in P_{2} }} ,w_{2} = - { }\gamma e + \left( {\mu + \delta } \right)v \le 0$$

$$f\left( {y\left( t \right)} \right)| _{{y\left( t \right) \in P_{3} }} ,w_{3} = - { }\beta sv + \left( {\mu + \gamma } \right)e \le 0$$

$$f\left( {y\left( t \right)} \right)| _{{y\left( t \right) \in P_{4} }} ,w_{4} = \beta sv + \mu s - \mu - \alpha z \le 0$$

$$f\left( {y\left( t \right)} \right)| _{{y\left( t \right) \in P_{5} }} ,w_{5} = 0$$

This shows that all solutions of the system (3) entered in $\Phi$. Hence the feasible region $\Phi$ is positively invariant which means the system (3) is both mathematically and biologically well posed in $\Phi$^30^.

## Qualitative investigation of the deterministic model

### Animosity-free equilibrium point

In the absence of animosity towards mathematics the time independent solution of the system (3) is said to be the animosity-free equilibrium point denoted by ${E}^{0}$ and after some steps of computations we have got ${E}^{0}=\left({s}^{0}, {e}^{0}, {v}^{0}, {z}^{0}\right)=\left(1, 0, 0, 0\right)$.

### Basic reproduction number

Take $X={(s, e, v, z)}^{T},$ and system (3) rewritten as

$$\frac{dX}{{dt}} = f_{i} - v_{i} ,$$

where

$$f_{i} = \left[ {\begin{array}{*{20}c} {\beta sv} \\ 0 \\ \end{array} } \right],$$

$$f = \left( {\begin{array}{*{20}c} 0 & \beta & 0 \\ 0 & 0 & 0 \\ 0 & 0 & 0 \\ \end{array} } \right),\;v_{i} ^{ + } \left( x \right) = \left( {\begin{array}{*{20}c} 0 \\ {\gamma e} \\ {\delta v} \\ \end{array} } \right),\;v_{i} ^{ - } \left( x \right) = \left( {\begin{array}{*{20}c} {\left( {\mu + \gamma } \right)e} \\ {\left( {\mu + \delta } \right)v} \\ {\left( {\mu + \alpha } \right)z} \\ \end{array} } \right),\;{\text{and}}$$

$$v_{i}^{ - } \left( x \right) - v_{i}^{ + } \left( x \right) = {\varvec{v}}_{{\varvec{i}}} = \left( {\begin{array}{*{20}c} {\left( {\mu + \gamma } \right)e} \\ {\left( {\mu + \delta } \right)v - \gamma e} \\ {\left( {\mu + \alpha } \right)z - \delta v} \\ \end{array} } \right).$$

Then by applying Mathematica we have got

$${\text{v}} = \left( {\begin{array}{*{20}c} {\mu + \gamma } & 0 & 0 \\ { - \gamma } & {\mu + \delta } & 0 \\ 0 & { - \delta } & {\mu + \alpha } \\ \end{array} } \right)$$

$$v^{( - 1)} = \left( {\begin{array}{*{20}c} {\frac{1}{{\left( {\mu + \gamma } \right)}}} & 0 & 0 \\ {\frac{\gamma }{{\left( {\mu + \gamma } \right)\left( {\mu + \delta } \right)}}} & {\frac{1}{{\left( {\mu + \delta } \right)}}} & 0 \\ {\frac{\gamma \delta }{{\left( {\mu + \gamma } \right)\left( {\mu + \delta } \right)\left( {\mu + \alpha } \right) }}} & {\frac{\delta }{{\left( {\mu + \delta } \right)\left( {\mu + \alpha } \right)}}} & {\frac{1}{{\left( {\mu + \alpha } \right)}}} \\ \end{array} } \right)$$

and

$$fv^{ - 1} = \left( {\begin{array}{*{20}c} {\frac{\beta \gamma }{{\left( {\mu + \gamma } \right)\left( {\mu + \delta } \right)}}} & {\frac{\beta }{{\left( {\mu + \delta } \right)}}} & 0 \\ 0 & 0 & 0 \\ 0 & 0 & 0 \\ \end{array} } \right).$$

Then the largest spectral radius of $f{v}^{-1}$ is the basic reproduction number of system (3) denoted by ${\mathcal{R}}_{0}$ which is given by ${\mathcal{R}}_{0}=\frac{\beta \gamma }{(\mu +\gamma )(\mu +\delta )}.$

### Animosity-Dominance equilibrium point

In the presence of animosity towards mathematics, the time dependent solution of the system (3^3^) is said to be animosity-dominance equilibrium point denoted by ${E}^{*}$ given by ${E}^{*}=\left({s}^{*}, {e}^{*}, {v}^{*}, {z}^{*}\right)$ where after some steps of calculations we have got

$$s^{*} = \frac{1}{{R_{0} }},$$

$$e^{*} = \frac{{\left( {\mu + \delta } \right)\left( {\frac{1}{{{\mathcal{R}}_{0} }} - 1} \right)\gamma \mu \left( {\mu + \alpha } \right)}}{{\gamma \left( {\alpha \delta \gamma - \left( {\mu + \alpha } \right)\left( {\mu + \delta } \right)\left( {\mu + \gamma } \right)} \right)}},$$

$$v^{*} = \frac{{\left( {\frac{1}{{{\mathcal{R}}_{0} }} - 1} \right)\gamma \mu \left( {\mu + \alpha } \right)}}{{\left( {\alpha \delta \gamma - \left( {\mu + \alpha } \right)\left( {\mu + \delta } \right)\left( {\mu + \gamma } \right)} \right)}},$$

$$z^{*} = \frac{{\delta \left( {\frac{1}{{{\mathcal{R}}_{0} }} - 1} \right)\gamma \mu \left( {\mu + \alpha } \right)}}{{\left( {\mu + \alpha } \right)\left( {\alpha \delta \gamma - \left( {\mu + \alpha } \right)\left( {\mu + \delta } \right)\left( {\mu + \gamma } \right)} \right)}}$$

Note: The animosity-dominance equilibrium point ${E}^{*}$ is exists when ${\mathcal{R}}_{0}\ge 1$.

### Stability analysis of equilibrium points

#### Theorem 3.1

*Routh-Hurwitz Criteria *^13,23^.

Suppose the characteristic polynomial of the matrix A is given by ${P}_{A}\left(\lambda \right)=\mathrm{det}\left(\lambda I-A\right)={\lambda }^{n}+{a}_{1}{\lambda }^{n-1}+\dots ..+{a}_{n}$ and define $k$ matrices as follows:

$$H_{1} = \left( {a_{1} } \right),H_{2} = \left( {\begin{array}{*{20}c} {a_{1} } & 1 \\ {a_{3} } & {a_{2} } \\ \end{array} } \right),H_{3} = \left( {\begin{array}{*{20}c} {a_{1} } & 1 & 0 \\ {a_{3} } & {a_{2} } & {a_{1} } \\ {a_{5} } & {a_{4} } & {a_{3} } \\ \end{array} } \right) ,$$

$$H_{j} = \left( {\begin{array}{*{20}c} {a_{1} } & 1 & 0 & 0 & \ldots & 0 \\ {a_{3} } & {a_{2} } & {a_{1} } & 1 & \ldots & 0 \\ {a_{5} } & {a_{4} } & {a_{3} } & {a_{2} } & \ldots & 0 \\ {a_{2j - 1} } & {a_{2j - 2} } & {a_{2j - 3} } & {a_{2j - 4} } & \ldots & {a_{j} } \\ \end{array} } \right),H_{k} = \left( {\begin{array}{*{20}c} {a_{1} } & 1 & 0 & \ldots & 0 \\ {a_{3} } & {a_{2} } & {a_{1} } & \ldots & 0 \\ . & . & . & \ldots & 0 \\ . & . & . & & {..} \\ 0 & 0 & {..} & & {a_{j} } \\ \end{array} } \right),$$

where the $(l, m)$ term in the matrix ${H}_{j}$ is ${a}_{2l-1}$ for $0<2l-m<k,$1 for $2l<m$, $0$ for $2l<m \, or \, 2l>k+m.$

Then all eigenvalues have negative real parts, that is, the steady-state is stable if and only the determinants of all Hurwitz matrices are positive $et{H}_{j}>0$ $j=1, 2, 3, .. k$.

To show the local stability use linearization principle and Lyapunov function for the global stability of the system (3) equilibrium points respectively. Linearity of the system (3) determined by the help of Jacobean matrix is given by

$$J\left( {s, e, v, z} \right) = \left( {\begin{array}{*{20}c} { - \mu - \beta v} & 0 & { - \beta s} & \alpha \\ {\beta v} & { - \left( {\mu + \gamma } \right)} & {\beta s} & 0 \\ 0 & \gamma & { - \left( {\mu + \delta } \right)} & 0 \\ 0 & 0 & \delta & { - \left( {\mu + \alpha } \right)} \\ \end{array} } \right).$$

#### Theorem 3.2

The animosity-free equilibrium point is locally asymptotically stable if ${\mathcal{R}}_{0}<1.$

#### *Proof*

The Jacobean matrix of the system (3) at the animosity-free equilibrium point is.

4 $$J\left( {1 ,{ }0,{ }0,{ }0} \right) = \left( {\begin{array}{*{20}c} { - \mu } & 0 & \alpha & 0 \\ 0 & { - \left( {\mu + \gamma } \right)} & \beta & 0 \\ 0 & \gamma & { - \left( {\mu + \delta } \right)} & 0 \\ 0 & 0 & \delta & { - \left( {\mu + \alpha } \right)} \\ \end{array} } \right)$$

From the Jacobean matrix, the characteristics equation is obtained as

$$\left| {\begin{array}{*{20}c} { - \mu - \lambda { }} & 0 & \alpha & 0 \\ 0 & { - \left( {\mu + \gamma } \right) - \lambda { }} & \beta & 0 \\ 0 & \gamma & { - \left( {\mu + \delta } \right) - \lambda { }} & 0 \\ 0 & 0 & \delta & { - \left( {\mu + \alpha } \right) - \lambda { }} \\ \end{array} } \right| = 0.$$

$$\Rightarrow \left( { - \mu - \lambda } \right)\left[ {\left( { - \left( {\mu + \gamma } \right) - \lambda } \right)\left( { - \left( {\mu + \delta } \right) - \lambda } \right)\left( { - \left( {\mu + \alpha } \right) - \lambda } \right) - \beta \gamma \left( { - \left( {\mu + \alpha } \right) - \lambda } \right)} \right] = 0.$$

$$\Rightarrow \left( { - \mu - \lambda } \right)\left( { - \left( {\mu + \alpha } \right) - \lambda } \right)\left[ {\left( { - \left( {\mu + \gamma } \right) - \lambda } \right)\left( { - \left( {\mu + \delta } \right) - \lambda } \right) - \beta \gamma } \right] = 0.$$

$$\Rightarrow \left( { - \mu - \lambda } \right)\left( { - \left( {\mu + \alpha } \right) - \lambda } \right)\left[ {\lambda^{2} + \left( {\left( {\mu + \gamma } \right) + \left( {\mu + \delta } \right)} \right)\lambda + \left( {\mu + \gamma } \right)\left( {\mu + \delta } \right) - \beta \gamma } \right] = 0.$$

$$\Rightarrow \left( { - \mu - \lambda } \right)\left( { - \left( {\mu + \alpha } \right) - \lambda } \right)\left[ {\lambda^{2} + \left( {\left( {\mu + \gamma } \right) + \left( {\mu + \delta } \right)} \right)\lambda + \left( {\mu + \gamma } \right)\left( {\mu + \delta } \right)\left( {1 - {\mathcal{R}}_{0} } \right)} \right] = 0.$$

Clearly from $\left(-\mu -\lambda \right)\left(-\left(\mu +\alpha \right)-\lambda \right),$ we obtained ${\lambda }_{1}=-\mu$,${\lambda }_{2}=-\left(\mu +\alpha \right)$ are real, negative and distinct. After some calculations the remaining two eigenvalues from the quadratic equation.

${\lambda }^{2}+\left(\left(\mu +\alpha \right)+\left(\mu +\delta \right)\right)\lambda +\left(\mu +\alpha \right)\left(\mu +\delta \right)\left(1-{\mathcal{R}}_{0}\right)=0$, are real, negative and distinct if ${\mathcal{R}}_{0}<1$. Thus, the animosity-free equilibrium point ${E}^{0}$ is locally asymptotically stable whenever ${\mathcal{R}}_{0}<1$ otherwise it is unstable.$\square$

#### Theorem 3.3

The animosity-dominance equilibrium point is locally asymptotically stable if ${\mathcal{R}}_{0}>1$.

#### *Proof*

The Jacobean matrix of the system (3) at the animosity-dominance equilibrium ${E}^{*}$ point is.

5 $$J\left( {s^{*} , e^{*} , v^{*} , z^{*} } \right) = \left( {\begin{array}{*{20}c} { - \beta v^{*} - \mu } & 0 & { - \beta s^{*} } & \alpha \\ {\beta v^{*} } & { - \left( {\mu + \gamma } \right)} & {\beta s^{*} } & 0 \\ 0 & \gamma & { - \left( {\mu + \delta } \right)} & 0 \\ 0 & 0 & \delta & { - \left( {\mu + \alpha } \right)} \\ \end{array} } \right)$$

Then the eigenvalue of the system (5) is obtained from the characteristics equation

$$\left| {\begin{array}{*{20}c} { - \left( {\beta v^{*} + \mu } \right) - \lambda } & 0 & { - \beta s^{*} } & \alpha \\ {\beta v^{*} } & { - \left( {\mu + \gamma } \right) - \lambda } & {\beta s^{*} } & 0 \\ 0 & \gamma & { - \left( {\mu + \delta } \right) - \lambda } & 0 \\ 0 & 0 & \delta & { - \left( {\mu + \alpha } \right) - \lambda } \\ \end{array} } \right| = 0$$

The characteristics polynomial after simple simplification is

$$\begin{aligned} p\left( \lambda \right) = & \lambda^{4} + \left[ {\left( {\left( {\mu + \beta v^{*} } \right) + \left( {\mu + \alpha } \right)} \right) + \left( {\left( {\mu + \gamma } \right) + \left( {\mu + \delta } \right)} \right)} \right]\lambda^{3} \\ & + \left[ {\left( {\left( {\mu + \beta v^{*} } \right) + \left( {\mu + \alpha } \right)} \right)\left( {\left( {\mu + \gamma } \right) + \left( {\mu + \delta } \right)} \right) + \left( {\mu + \beta v^{*} } \right)\left( {\mu + \alpha } \right)} \right]\lambda^{2} \\ & + \left[ {\left( {\mu + \beta v^{*} } \right)\left( {\mu + \alpha } \right)\left( {\left( {\mu + \gamma } \right) + \left( {\mu + \delta } \right)} \right)} \right]\lambda + \beta s^{*} \beta v^{*} \gamma \left( {\left( {\mu + \alpha } \right) + \lambda } \right) + \alpha \beta v^{*} \gamma \delta . \\ \end{aligned}$$

6 $$p\left( \lambda \right) = a_{4} \lambda^{4} + a_{3} \lambda^{3} + a_{2} \lambda^{2} + a_{1} \lambda + a_{0} .$$

where

$$a_{4} = 1,$$

$$a_{3} = \left( {\left( {\mu + \beta v^{*} } \right) + \left( {\mu + \alpha } \right)} \right) + \left( {\left( {\mu + \gamma } \right) + \left( {\mu + \delta } \right)} \right),$$

$$a_{2} = \left( {\left( {\mu + \beta v^{*} } \right) + \left( {\mu + \alpha } \right)} \right)\left( {\left( {\mu + \gamma } \right) + \left( {\mu + \delta } \right)} \right) + \left( {\mu + \beta v^{*} } \right)\left( {\mu + \alpha } \right),$$

$$a_{1} = \left( {\mu + \beta v^{*} } \right)\left( {\mu + \alpha } \right)\left( {\left( {\mu + \gamma } \right) + \left( {\mu + \delta } \right)} \right),$$

$$a_{0} = s^{*} \beta v^{*} \gamma \left( {\left( {\mu + \alpha } \right) + \lambda } \right) + \alpha \beta v^{*} \gamma \delta ,$$

$$s^{*} = \frac{1}{{R_{0} }},$$

$$v^{*} = \frac{{\left( {\frac{1}{{{\mathcal{R}}_{0} }} - 1} \right)\gamma \mu \left( {\mu + \alpha } \right)}}{{\left( {\alpha \delta \gamma - \left( {\mu + \alpha } \right)\left( {\mu + \delta } \right)\left( {\mu + \gamma } \right)} \right)}}.$$

By Routh–Hurwitz stability criteria Theorem 3.1 above or by referring^13,23^, all eigenvalues of (6) have negative real parts see in^17,29^ whenever ${\mathcal{R}}_{0}>1$. Hence whenever ${\mathcal{R}}_{0}>1,$ then the animosity-dominance equilibrium point is locally asymptotically stable.$\square$

#### Theorem 3.4

The animosity-free equilibrium point is globally stable if ${\mathcal{R}}_{0}<1.$

#### *Proof*

Consider the Lyapunov function $v\left(a, z\right)=me+na$, where $m=\frac{\beta \gamma }{(\mu +\gamma )(\mu +\delta )}$, and $n=\frac{\beta }{(\mu +\delta )}$,

$$l\left( {e, v} \right) = me + nv = \frac{\beta \gamma }{{\left( {\mu + \gamma } \right)\left( {\mu + \delta } \right)}}e + \frac{\beta }{{\left( {\mu + \delta } \right)}}v,$$ ,

$$\Rightarrow l\left( {e,v} \right) = \frac{\beta \gamma }{{\left( {\mu + \gamma } \right)\left( {\mu + \delta } \right)}}e + \frac{\beta }{{\left( {\mu + \delta } \right)}}v$$

$$\Rightarrow \frac{dl}{{dt}} = \frac{\beta \gamma }{{\left( {\mu + \gamma } \right)\left( {\mu + \delta } \right)}}\left( {\beta sv - \left( {\mu + \gamma } \right)e} \right) + \frac{\beta }{{\left( {\mu + \delta } \right)}}\left( {\left( {\gamma e - \left( {\mu + \delta } \right)v} \right)} \right)$$

$$\Rightarrow \frac{dl}{{dt}} = \frac{\beta \beta v\gamma }{{\left( {\mu + \gamma } \right)\left( {\mu + \delta } \right)}} - \frac{{\beta \gamma \left( {\mu + \gamma } \right)e}}{{\left( {\mu + \gamma } \right)\left( {\mu + \delta } \right)}} + \frac{\beta \gamma e}{{\left( {\mu + \delta } \right)}} - \frac{{\beta \left( {\mu + \delta } \right)v}}{{\left( {\mu + \delta } \right)}}$$

$$\Rightarrow \frac{dl}{{dt}} = \left( {\frac{\beta \beta \gamma }{{\left( {\mu + \gamma } \right)\left( {\mu + \delta } \right)}} - \frac{{\beta \left( {\mu + \delta } \right)}}{{\left( {\mu + \delta } \right)}}} \right)v + \left( {\frac{\beta \gamma }{{\left( {\mu + \delta } \right)}} - \frac{{\beta \gamma \left( {\mu + \gamma } \right)}}{{\left( {\mu + \gamma } \right)\left( {\mu + \delta } \right)}}} \right)e$$

$$\Rightarrow \frac{dl}{{dt}} = \beta \left( {\frac{\beta \gamma }{{\left( {\mu + \gamma } \right)\left( {\mu + \delta } \right)}} - 1} \right)v + \left( {\frac{\beta \gamma }{{\left( {\mu + \delta } \right)}} - \frac{\beta \gamma }{{\left( {\mu + \delta } \right)}}} \right)e$$

$$\Rightarrow \frac{dl}{{dt}} = \beta \left( {\frac{\beta \gamma }{{\left( {\mu + \gamma } \right)\left( {\mu + \delta } \right)}} - 1} \right)a$$

$$\Rightarrow \frac{dl}{{dt}} = \beta \left( {{\mathcal{R}}_{0} - 1} \right)v$$

Thus $\frac{dl}{dt}<0$, if ${\mathcal{R}}_{0}<1$ and the equality $\frac{dv}{dt}=0$ holds if $v=0$ and hence according LaSalle’s invariant principle used in^30^ the model animosity-free equilibrium point ${E}^{0}$ is globally asymptotically stable when ${\mathcal{R}}_{0}<1$.

### Global stability analysis of animosity-dominance equilibrium

The aim of this section is to investigate the global stability of ${E}^{*}$ in the positively invariant set $\Phi$ with the aid of Volterra–Lyapunov stable matrices. To organize this, we express the Lyapunov function as:

7 $$L\left( {s ,e ,v ,z} \right) = v_{1} \left( {s - s^{*} } \right)^{2} + v_{2} \left( {e - e^{*} } \right)^{2} + v_{3} \left( {v - v^{*} } \right)^{2} + v_{4} \left( {z - z^{*} } \right)^{2}$$

where ${v}_{1}, {v}_{2},{v}_{3},{v}_{4}$ are positive constant. The time derivative of $L\left(s ,e ,v ,z\right)$ belongs the solution of the system (3) is

$$\begin{gathered} \frac{dL}{{dt}} = 2v_{1} \left( {s - s^{*} } \right)\left[ {\alpha \left( {z - z^{*} } \right) - \mu \left( {s - s^{*} } \right) - \beta sv + \beta s^{*} v^{*} } \right] + 2v_{2} \left( {e - e^{*} } \right)\left[ {\beta sv - \beta s^{*} v^{*} - \left( {\mu + \gamma } \right)\left( {e - e^{*} } \right)} \right] \hfill \\ + 2v_{3} \left( {v - v^{*} } \right)\left[ {\gamma \left( {e - e^{*} } \right) - \left( {\mu + \delta } \right)v} \right] + 2v_{4} \left( {z - z^{*} } \right)\left[ {\delta \left( {v - v^{*} } \right) - \left( {\mu + \alpha } \right)\left( {z - z^{*} } \right)} \right]. \hfill \\ \end{gathered}$$

By adding and subtracting the expression $\beta {s}^{*}v$ in the first and second closed bracket, we obtain

8 $$\begin{aligned} \frac{dL}{{dt}} = & - 2v_{1} \left( {\mu + \beta v} \right)\left( {s - s^{*} } \right)^{2} - 2v_{1} \beta s^{*} \left( {s - s^{*} } \right)\left( {v - v^{*} } \right) + 2v_{1} \alpha \left( {s - s^{*} } \right)\left( {z - z^{*} } \right) + 2v_{2} \beta v\left( {s - s^{*} } \right)\left( {e - e^{*} } \right) \\ & - 2v_{2} \beta s^{*} \left( {e - e^{*} } \right)\left( {v - v^{*} } \right) - 2v_{2} \left( {\mu + \gamma } \right)\left( {e - e^{*} } \right)^{2} + 2v_{3} \gamma \left( {v - v^{*} } \right)\left( {e - e^{*} } \right) - 2v_{3} \left( {\mu + \delta } \right)\left( {v - v^{*} } \right)^{2} \\ & + 2v_{4} \delta \left( {z - z^{*} } \right)\left( {v - v^{*} } \right) - 2v_{4} \left( {\mu + \alpha } \right)\left( {z - z^{*} } \right)^{2} = M\left( {VA + A^{T} V^{T} } \right)M^{T} \\ \end{aligned}$$

where

$$M = (s - s^{*} ,e - e^{*} ,v - v^{*} ,z - z^{*} ),$$

$$V=diag({v}_{1}, {v}_{2}, { v}_{3}, { v}_{4})$$ , and

9 $$A = \left( {\begin{array}{*{20}c} { - \left( {\mu + \beta v} \right)} & 0 & { - \beta } & \alpha \\ {\beta v} & { - \left( {\mu + \gamma } \right)} & { - \beta s^{*} } & 0 \\ 0 & \gamma & { - \left( {\mu + \delta } \right)} & 0 \\ 0 & 0 & \delta & { - \left( {\mu + \alpha } \right)} \\ \end{array} } \right)$$

To establish the global stability of the animosity-dominance equilibrium point ${E}^{*}$, we investigate the matrix $A$ defined in Eq. (9) is Volterra–Lyapunov stable. We concisely explain the following basic definitions related to Volterra–Lyapunov stable matrices stated in^6^.

Let ${A}_{nxn}$ be a real matrices then.

- (D1) All the eigenvalues of A have negative (positive) real parts if and only if there exists a matrix $H > 0$ such that ${A}^{T}{B}^{T}>0$ $< 0(> 0)$.
  (D2) The nonsingular matrix ${A}_{nxn}$ is Volterra–Lyapunov stable if there exists a positive diagonal $n \times n$ matrix V such that $A+{A}^{T}{V}^{T}$ $< 0$.
  (D3) The nonsingular matrix ${A}_{nxn}$ is diagonal stable if there exists a positive diagonal $n \times n$ matrix V such that $VA+{A}^{T}{V}^{T}>0.$

#### Lemma 2

The matrix $$A=\left[\begin{array}{cc}{a}_{11}& {a}_{12}\\ {a}_{21}& {a}_{22}\end{array}\right] ,$$ is Volterra–Lyapunov stable if and only if:

- i. $${a}_{11}<0,$$
- ii. $${a}_{22}<0,$$

#### Lemma 3

Consider the nonsingular matrix ${A}_{nxn}=\left[{a}_{ij}\right],$ $n\ge 2$, ${V}_{nxn}=diag\left({v}_{1}, {v}_{2}, .. {v}_{n}\right)$, and $C={A}^{-1}$, such that.

- i. $${a}_{nn}>0,$$
- ii. $$\tilde{V }\tilde{A }+{(\tilde{V }\tilde{A })}^{T}>0,$$
- iii. $$\tilde{V }\tilde{C }+{(\tilde{V }\tilde{C })}^{T}>0>0$$

Now choose ${v}_{n}>0$, such that $VA+{A}^{T}{V}^{T}>0$.

Note: $\tilde{A }$ is the $(n-1)x(n-1)$ matrix, obtained by deleting the last row and column of the matrix ${A}_{nxn}.$

#### Theorem 3.5

The square matrix $A$ defined in (9) is Volterra–Lyapunov stable.

#### *Proof*

Clearly ${A}_{44}>0$, and the matrix when we delete last row and column of matrix $-A$ is defined as:

10 $$M = - \tilde{A} = \left( {\begin{array}{*{20}c} {\left( {\mu + \beta v} \right)} & 0 & \beta \\ { - \beta v} & {\left( {\mu + \gamma } \right)} & {\beta s^{*} } \\ 0 & { - \gamma } & {\left( {\mu + \delta } \right)} \\ \end{array} } \right)$$

Based on Lemma 3, we state and prove that $=-\tilde{A }$ , and $C=-\stackrel{\sim }{{A}^{-1}}$ are diagonal stable in the following conditions to satisfied Lemma 3. Hence the matrix A is Volterra–Lyapunov stable.

#### Condition 1

The square matrix $M$ defined in (10) is diagonal stable.

#### *Proof*

The diagonal stability of $M$, is guaranteed by the following steps:

Step 1. It is clear that $-{\tilde{A }}_{33}=\left(\mu +\delta \right)>0$.
Step 2. By using Lemma 2, we need to show $\tilde{M }$ is diagonal stable. From Eq. (10), we obtain

$$\tilde{M }=\left(\begin{array}{cc}\left(\mu +\beta v\right)& 0\\ -\beta v& \left(\mu +\gamma \right)\end{array}\right)$$

Clearly ${\tilde{M }}_{11}>0, {\tilde{M }}_{22}>0$, and $\mathrm{det}\left(\tilde{M }\right)=\left(\mu +\beta v\right)\left(\mu +\gamma \right)>0$. So $\tilde{M }$ is diagonal stable.

Step 3. We show that $\stackrel{\sim }{{M}^{-1}}$ is diagonal stable .The square matrix $\stackrel{\sim }{{M}^{-1}}$ after simplifications ;

$$\stackrel{\sim }{{M}^{-1}}=\left(\begin{array}{cc}\frac{\left(\mu +\gamma \right)\left(\mu +\delta \right)+\beta \gamma {s}^{*}}{\left(\mu +\beta v\right)\left[\left(\mu +\gamma \right)\left(\mu +\delta \right)+\beta \gamma {s}^{*}\right]+\beta \gamma \beta v}& \frac{-\beta \gamma }{\left(\mu +\beta v\right)\left[\left(\mu +\gamma \right)\left(\mu +\delta \right)+\beta \gamma {s}^{*}\right]+\beta \gamma \beta v}\\ \frac{\beta v\left(\mu +\delta \right)}{\left(\mu +\beta v\right)\left[\left(\mu +\gamma \right)\left(\mu +\delta \right)+\beta \gamma {s}^{*}\right]+\beta \gamma \beta v}& \frac{\left(\mu +\beta v\right)\left(\mu +\delta \right)}{\left(\mu +\beta v\right)\left[\left(\mu +\gamma \right)\left(\mu +\delta \right)+\beta \gamma {s}^{*}\right]+\beta \gamma \beta v}\end{array}\right)$$

It is easy to show that.

$$\stackrel{\sim }{{M}_{11}^{-1}}=>0,\stackrel{\sim }{{ M}_{22}^{-1}}=>0, \, \text{and} \, \mathrm{det}\left(\stackrel{\sim }{{M}^{-1}}\right)>0.$$

Hence $\stackrel{\sim }{{M}^{-1}}$ is diagonal stable. Therefore in this condition we have proved that $M=-\tilde{A }$ is diagonal stable.

#### Condition 2

The matrix $C=-\stackrel{\sim }{{A}^{-1}}$ is diagonal stable.

#### *Proof*

The matrix $-\stackrel{\sim }{{A}^{-1}}$ which obtained after a simple simplification is.

$$- \tilde{A}^{ - 1} = \left( {\begin{array}{*{20}c} {a_{11} } & {a_{12} } & {a_{13} } \\ {a_{21} } & {a_{22} } & {a_{23} } \\ {a_{31} } & {a_{32} } & {a_{33} } \\ \end{array} } \right),$$

where

$${a}_{11}=\frac{\left(\mu +\gamma \right)\left(\mu +\delta \right)\left(\mu +\alpha \right)+\beta \gamma {s}^{*}\left(\mu +\alpha \right)}{\left(\mu +\beta v\right)\left(\mu +\gamma \right)\left(\mu +\delta \right)\left(\mu +\alpha \right)+\beta \gamma {s}^{*}\left(\mu +\alpha \right)+\beta v\beta \gamma \left(\mu +\alpha \right)-\alpha \beta v\gamma \delta }$$

$${a}_{12}=\frac{\alpha \gamma \delta -\beta \gamma \left(\mu +\alpha \right) }{\left(\mu +\beta v\right)\left(\mu +\gamma \right)\left(\mu +\delta \right)\left(\mu +\alpha \right)+\beta \gamma {s}^{*}\left(\mu +\alpha \right)+\beta v\beta \gamma \left(\mu +\alpha \right)-\alpha \beta v\gamma \delta }$$

$${a}_{13}=\frac{-\beta \left(\mu +\gamma \right)\left(\mu +\alpha \right)+\alpha \delta \left(\mu +\gamma \right) }{\left(\mu +\beta v\right)\left(\mu +\gamma \right)\left(\mu +\delta \right)\left(\mu +\alpha \right)+\beta \gamma {s}^{*}\left(\mu +\alpha \right)+\beta v\beta \gamma \left(\mu +\alpha \right)-\alpha \beta v\gamma \delta }$$

$${a}_{21}=\frac{-[\left(\mu +\gamma \right)\left(\mu +\delta \right)\left(\mu +\alpha \right)+\left(\mu +\alpha \right)\gamma \beta {s}^{*}]}{\left(\mu +\beta v\right)\left(\mu +\gamma \right)\left(\mu +\delta \right)\left(\mu +\alpha \right)+\beta \gamma {s}^{*}\left(\mu +\alpha \right)+\beta v\beta \gamma \left(\mu +\alpha \right)-\alpha \beta v\gamma \delta }$$

$${a}_{22}=\frac{\left(\mu +\beta v\right)\left(\mu +\delta \right)\left(\mu +\alpha \right)-\beta \left(\mu +\beta v\right)\left(\mu +\alpha \right)+\alpha \delta \left(\mu +\beta v\right)}{\left(\mu +\beta v\right)\left(\mu +\gamma \right)\left(\mu +\delta \right)\left(\mu +\alpha \right)+\beta \gamma {s}^{*}\left(\mu +\alpha \right)+\beta v\beta \gamma \left(\mu +\alpha \right)-\alpha \beta v\gamma \delta }$$

$${a}_{23}=\frac{-\beta {s}^{*}\left(\mu +\beta v\right)\left(\mu +\alpha \right)-\beta v\beta \left(\mu +\alpha \right)+\alpha \beta v\delta }{\left(\mu +\beta v\right)\left(\mu +\gamma \right)\left(\mu +\delta \right)\left(\mu +\alpha \right)+\beta \gamma {s}^{*}\left(\mu +\alpha \right)+\beta v\beta \gamma \left(\mu +\alpha \right)-\alpha \beta v\gamma \delta }$$

$${a}_{31}=\frac{\beta \gamma v\left(\mu +\alpha \right) }{\left(\mu +\beta v\right)\left(\mu +\gamma \right)\left(\mu +\delta \right)\left(\mu +\alpha \right)+\beta \gamma {s}^{*}\left(\mu +\alpha \right)+\beta v\beta \gamma \left(\mu +\alpha \right)-\alpha \beta v\gamma \delta }$$

$${a}_{32}=\frac{\gamma \left(\mu +\beta v\right)\left(\mu +\alpha \right)}{\left(\mu +\beta v\right)\left(\mu +\gamma \right)\left(\mu +\delta \right)\left(\mu +\alpha \right)+\beta \gamma {s}^{*}\left(\mu +\alpha \right)+\beta v\beta \gamma \left(\mu +\alpha \right)-\alpha \beta v\gamma \delta }$$

$${a}_{33}=\frac{\left(\mu +\beta v\right)\left(\mu +\gamma \right)\left(\mu +\alpha \right) }{\left(\mu +\beta v\right)\left(\mu +\gamma \right)\left(\mu +\delta \right)\left(\mu +\alpha \right)+\beta \gamma {s}^{*}\left(\mu +\alpha \right)+\beta v\beta \gamma \left(\mu +\alpha \right)-\alpha \beta v\gamma \delta }$$

Clearly ${a}_{33}>0,$ then we need to show $\tilde{C }$ is diagonal stable.

$$\tilde{C} = \left( {\begin{array}{*{20}c} {a_{11} } & {a_{12} } \\ {a_{21} } & {a_{22} } \\ \end{array} } \right),$$

$${a}_{11}=\frac{\left(\mu +\gamma \right)\left(\mu +\delta \right)\left(\mu +\alpha \right)+\beta \gamma {s}^{*}\left(\mu +\alpha \right)}{\left(\mu +\beta v\right)\left(\mu +\gamma \right)\left(\mu +\delta \right)\left(\mu +\alpha \right)+\beta \gamma {s}^{*}\left(\mu +\alpha \right)+\beta v\beta \gamma \left(\mu +\alpha \right)-\alpha \beta v\gamma \delta }>0,$$

$${a}_{22}=\frac{\left(\mu +\beta v\right)\left(\mu +\delta \right)\left(\mu +\alpha \right)-\beta \left(\mu +\beta v\right)\left(\mu +\alpha \right)+\alpha \delta \left(\mu +\beta v\right)}{\left(\mu +\beta v\right)\left(\mu +\gamma \right)\left(\mu +\delta \right)\left(\mu +\alpha \right)+\beta \gamma {s}^{*}\left(\mu +\alpha \right)+\beta v\beta \gamma \left(\mu +\alpha \right)-\alpha \beta v\gamma \delta }>0,$$

$$\mathrm{det}\left(\tilde{C }\right)={a}_{11}{a}_{22}-{a}_{21}{a}_{12}={a}_{11}{a}_{22}+{a}_{21}{a}_{12}>0, \text{since} \, {a}_{21}<0.$$

Hence $\tilde{C }$ is diagonal stable. Therefore from condition 2 we have proved $C=-\stackrel{\sim }{{A}^{-1}}$ is diagonal stable. Finally from condition 1 and 2 we conclude $M=-\tilde{A }$ and $C=-\stackrel{\sim }{{A}^{-1}}$ are diagonal stable. Then finally due to the above basic definition, Lemmas and conditions, we have the following conclusions for the globally stability of the animosity-dominance equilibrium.

#### Theorem 3.6

If ${R}_{0}>1$, then the animosity-dominance equilibrium point ${E}^{*}$ of the system (3) is globally stable in $\Phi$.

#### *Proof*

Lemmas 2 and 3 with the aid of Theorem 3.4 guaranteed that the animosity-dominance equilibrium of the system (3) is globally stable.

## Stochastic touch for SEATS Model (1)

### The transition probability

The transition probability is the probability of a stochastic process that transfer from state 1 to state 2. Here, the stochastic SEATS model consists of three random variables, i.e. $S(t),E(t),A(t). N(t)$ is the total number of students, which is assumed to be constant. Then $N(t) = N$ for all $t \ge 0$ where $N\left(t\right)= S\left(t\right)+ E(t)+A(t)+T(t)$. Thus, state variable $T(t)$ determined by rearrangement is given by $T\left(t\right)= N\left(t\right)- S\left(t\right)- E\left(t\right)- A\left(t\right)-T(t)$, where $t$ is time. Suppose an ordered pair $(S(t),E(t),A(t))=(s, e, a)$ and $(S\left(t + \Delta t\right),E\left(t + \Delta t\right),A\left(t + \Delta t\right)) = ({k}_{1}, {k}_{2}, {k}_{3})$ where *s*, e, a, ${k}_{1}$, ${k}_{2}$, ${k}_{3}$ = 0, 1, 2 …. Here the transition probability for the SEATS model can be constructed as:

$$\begin{gathered} Prob_{{\left( {k_{1} , k_{2} , k_{3} } \right), ))\left( {s, e, a} \right)}} \left( {t,t + \Delta t} \right) = Prob\left\{ {S\left( {t + \Delta t} \right) = k_{1} , E\left( {t + \Delta t} \right) = k_{2} , A\left( {t + \Delta t} \right) = k_{3} , \left( {S\left( t \right) = s,E\left( t \right) = e,A\left( t \right) = a} \right)} \right\} \hfill \\ = \left\{ {\begin{array}{*{20}l} {\left( {\mu N + \alpha T} \right){\Delta }t + o\left( {{\Delta }t} \right),\left( {k_{1} , k_{2} , k_{3} } \right) = \left( {s + 1,e,a} \right)} \hfill \\ {\left( {\beta SA} \right){\Delta }t + o\left( {{\Delta }t} \right),\left( {k_{1} , k_{2} , k_{3} } \right) = \left( {s - 1,e + 1,a} \right)} \hfill \\ {\mu S{\Delta }t + o\left( {{\Delta }t} \right),\left( {k_{1} , k_{2} , k_{3} } \right) = \left( {s - 1,e,a} \right)} \hfill \\ {\gamma E{\Delta }t + o\left( {{\Delta }t} \right),\left( {k_{1} , k_{2} , k_{3} } \right) = \left( {s,e - 1,a + 1} \right)} \hfill \\ {\mu E{\Delta }t + o\left( {{\Delta }t} \right),\left( {k_{1} , k_{2} , k_{3} } \right) = \left( {s,e - 1,a} \right)} \hfill \\ {\left( {\mu + \delta } \right)A{\Delta }t + o\left( {{\Delta }t} \right),\left( {k_{1} , k_{2} , k_{3} } \right) = \left( {s,e,a - 1} \right)} \hfill \\ {\left( {1 - \theta } \right){\Delta }t + o\left( {{\Delta }t} \right),\left( {k_{1} , k_{2} , k_{3} } \right) = \left( {s,e,a} \right)} \hfill \\ {o\left( {{\Delta }t} \right), otherwise} \hfill \\ \end{array} } \right. \hfill \\ \end{gathered}$$

where $$\theta =\mu N+\alpha T+\beta SA+\mu S+\gamma E+\mu E+\left(\mu +\delta \right)A.$$

The transition probabilities of susceptible, exposed, and animosity infected students in the time interval $(t+\Delta t)$ only depend on time $t$, at $t \ge 0$. The time value $\Delta t$ is assumed to be very small so that the change occurring in susceptible, exposed, animosity infected individuals is the maximum of one individual in such a short period time interval ∆*t*. The value of $o(\Delta t)$ represents a small probability value and satisfies $o(\Delta t) \Delta t = 0.$

### Animosity outbreak probability

Animosity outbreak issue occurs when the number of animosity infected students’ increases through time. The basic reproduction number $({\mathcal{R}}_{0})$ and the expected number of animosity infected students $(n)$ are used as a criterion for the occurrence of animosity outbreaks in the long term. The basic reproduction number $({\mathcal{R}}_{0})$ is the number of susceptible students getting animosity infected after an animosity infected student is introduced into the group. The basic reproduction number $({\mathcal{R}}_{0})$ has the same definition as the expected number of infected students $(n)$, which is calculated using a probability method. In deterministic models, animosity outbreak issues occur when ${\mathcal{R}}_{0}>1$, whereas in stochastic models this is associated with a condition when the expected number of animosity infected students $\left(n\right)>1$. The two quantities $({\mathcal{R}}_{0})$ and $n$ are obtained with different methods. The benchmark for the animosity outbreak probability is determined by the expected number of animosity infected individuals $\left(n\right)$, not from the basic reproduction number $({\mathcal{R}}_{0})$. Based on this process, the SEAS model has the following disease extinction probabilities:

$$Prob\left\{ {A\left( t \right) = 0} \right\} = \left\{ {\begin{array}{*{20}l} {1,} \hfill & {if\quad n \le 1} \hfill \\ {\tau ,} \hfill & {if\quad n > 1} \hfill \\ \end{array} } \right.$$ and as a result, the probability of animosity outbreak occurring is.

$$1 - Prob\left\{ {A\left( t \right) = 0} \right\} = \left\{ {\begin{array}{*{20}l} {0,} \hfill & {if\quad n \le 1} \hfill \\ {1 - \tau ,} \hfill & {if\quad n > 1} \hfill \\ \end{array} } \right.$$, where $\tau =\frac{N}{s{\mathcal{R}}_{0}}.$ Finally, in this study we considered large number of students for numerical simulation; for the remaining analysis part we have used the deterministic model rather than the stochastic touch.

## Optimal control analysis of the deterministic model

In this section, we formulated and analyzed a mathematical model with optimal control method^34^ to identify the best control strategy that reduces the number of animosity-infected individuals with minim total cost used during the interventions. The objective is to find the optimal values ${\mathfrak{u}}^{*}=\left({\mathfrak{u}}_{1}^{*}, {\mathfrak{u}}_{2}^{*}\right)$ of the controls $\mathfrak{u}=\left({\mathfrak{u}}_{1}, {\mathfrak{u}}_{2}\right)$ such that the associated state trajectories ${E}^{*}=\left({S}^{*},{E}^{*}, {A}^{*}, {T}^{*} \right)$ are solution of the system (1) in the intervention time interval $\left[0, {T}_{f}\right]$ with given initial conditions and minimize the objective functional. The control ${\mathfrak{u}}_{1}\left(t\right)$ represents the efforts on preventing animosity infection that helps to reduce contact rate of animosity, ${\mathfrak{u}}_{2}\left(t\right)$ is the control relates towards improvement of animosity recovery period. This is equated towards implementation of proper treatment and psychological counseling policies for animosity towards mathematics, so that recovery period can be improved by providing right treatment and counseling such that $0 \le {\mathfrak{u}}_{2}\left(t\right) \le 1$.

Then the system (1) is changed into

10 $$\begin{gathered} \frac{dS}{{dt}} = \mu N + \alpha T - \left( {1 - {\mathfrak{u}}_{1} } \right)\beta SA - \mu S, \hfill \\ \frac{dE}{{dt}} = \left( {1 - {\mathfrak{u}}_{1} } \right)\beta SA - \left( {\mu + \gamma } \right)E, \hfill \\ \frac{dA}{{dt}} = \gamma E - \left( {\mu + {\mathfrak{u}}_{2} \delta } \right)A, \hfill \\ \frac{dT}{{dt}} = {\mathfrak{u}}_{2} \delta A - \left( {\mu + \alpha } \right)T, \hfill \\ \end{gathered}$$

With the corresponding initial conditions

11 $$S\left( 0 \right) > 0,\left( 0 \right) \ge 0, A\left( 0 \right) \ge 0,\;{\text{and}}\;T\left( 0 \right) > 0$$

For this, our optimal control problem is to minimize the objective functional

12 $$J\left( {{\mathfrak{u}}_{1} , {\mathfrak{u}}_{2} } \right) = \mathop \smallint \limits_{0}^{{T_{f} }} \left( {{\mathfrak{w}}_{1} A + \frac{{{\mathfrak{B}}_{1} }}{2}{\mathfrak{u}}_{1}^{2} + \frac{{{\mathfrak{B}}_{2} }}{2}{\mathfrak{u}}_{2}^{2} } \right)dt$$

where $I\left(S, E, A, T,\mathfrak{u}\right) ={\mathfrak{w}}_{1}A+\frac{{\mathfrak{B}}_{1}}{2}{\mathfrak{u}}_{1}^{2}+\frac{{\mathfrak{B}}_{2}}{2}{\mathfrak{u}}_{2}^{2}$, measures the current cost at time t.

The coefficient ${\mathfrak{w}}_{1}$ is positive weight constant that characterizes the cost associated with minimizing the animosity infected individuals and $\frac{{\mathfrak{B}}_{1}}{2}$ and $\frac{{\mathfrak{B}}_{2}}{2}$ are the measures of relative costs of interventions associated with the controls ${\mathfrak{u}}_{1}$ and ${\mathfrak{u}}_{2}$, respectively, and also balances the units of integrand. In the cost functional, the term ${\mathfrak{w}}_{1}A$ refers the cost related to animosity infected class.

The set of admissible control functions is defined by

13 $${\Omega }_{{\mathfrak{u}}} = \left\{ {{\mathfrak{u}}_{1} \left( t \right), {\mathfrak{u}}_{2} \left( t \right) \in L^{2} :0 \le {\mathfrak{u}}_{1} \left( t \right), {\mathfrak{u}}_{2} \left( t \right) \le 1, t \in \left[ {0,T_{f} } \right]} \right\}$$

More precisely, we seek an optimal control pair

14 $$J\left( {{\mathfrak{u}}_{1}^{*} , {\mathfrak{u}}_{2}^{*} } \right) = \mathop {\min }\limits_{{{\Omega }_{s} }} J\left( {{\mathfrak{u}}_{1} , {\mathfrak{u}}_{2} } \right)$$

### Characterization of the optimal control

In this section, we present optimality conditions for the optimal control problem defined above and detail its properties. According to Pontryagin’s Maximum Principle in^4,13,37^, if ${\mathfrak{u}}^{*}\left(t\right)\in {\Omega }_{\mathfrak{u}}$ is optimal for dynamical system (10) with initial value (11) and (14) with fixed final time ${T}_{f}$, then there exists a non-trivial absolutely continuous mapping $\lambda :\left[0, {T}_{f}\right]\to {\mathbb{R}}^{4}$ $, \lambda =\left({\lambda }_{1}\left(t\right), {\lambda }_{2}\left(t\right), {\lambda }_{3}\left(t\right), {\lambda }_{4}\left(t\right)\right)$ called the adjoint vector, such that.

1. The Hamiltonian function is defined as
  15 $$\begin{aligned} {\mathcal{H}} = & {\mathfrak{w}}_{1} A + \frac{{{\mathfrak{B}}_{1} }}{2}{\mathfrak{u}}_{1}^{2} + \frac{{{\mathfrak{B}}_{2} }}{2}{\mathfrak{u}}_{2}^{2} + \lambda_{1} \left( {\mu N + \alpha T - \left( {1 - {\mathfrak{u}}_{1} } \right)\beta SA - \mu S} \right) \\ & + \lambda_{2} \left( {\left( {1 - {\mathfrak{u}}_{1} } \right)\beta SA - \left( {\mu + \gamma } \right)E} \right) \\ & + \lambda_{3} \left( {\gamma E - \left( {\mu + {\mathfrak{u}}_{2} \delta } \right)A} \right) + \lambda_{4} \left( {{\mathfrak{u}}_{2} \delta A - \left( {\mu + \alpha } \right)T} \right) \\ \end{aligned}$$
2. The control system is
  16 $$\frac{dS}{{dt}} = \frac{{\partial {\mathcal{H}}}}{{\partial \lambda_{1} }}, \frac{dE}{{dt}} = \frac{{\partial {\mathcal{H}}}}{{\partial \lambda_{2} }}, \frac{dA}{{dt}} = \frac{{\partial {\mathcal{H}}}}{{\partial \lambda_{3} }}, \frac{dT}{{dt}} = \frac{{\partial {\mathcal{H}}}}{{\partial \lambda_{4} }},$$
3. The adjoint system
  17 $$\frac{{d\lambda_{1} }}{dt} = - \frac{{\partial {\mathcal{H}}}}{\partial S}, \frac{{d\lambda_{2} }}{dt} = - \frac{{\partial {\mathcal{H}}}}{\partial E}, \frac{{d\lambda_{3} }}{dt} = - \frac{{\partial {\mathcal{H}}}}{\partial A}, \frac{{d\lambda_{4} }}{dt} = - \frac{{\partial {\mathcal{H}}}}{\partial T},$$
4. And the optimality condition is
  18 $${\mathcal{H}}\left( {{\mathbb{E}}^{*} ,{\mathfrak{u}},\lambda^{*} } \right) = \mathop {\min }\limits_{{{\mathfrak{u}} \in {\Omega }_{{\mathfrak{u}}} }} \left( {{\mathbb{E}}^{*} ,{\mathfrak{u}}^{*} ,\lambda^{*} } \right)$$
5. Moreover, the transversality condition is
  holds for almost all $t\in [0, {T}_{f}].$
  19 $$\lambda_{i} \left( {T_{f} } \right) = 0, i = 1, 2, 3, 4$$

also holds true. In the next result, we have discussed characterization of optimal controls and adjoint variables.

#### Theorem 5.1

Let ${\mathfrak{u}}^{*}= \left({\mathfrak{u}}_{1}^{*}, {\mathfrak{u}}_{2}^{*}\right)$ be the optimal control and $\left({S}^{*}(\cdot ),{E}^{*}(\cdot ), {A}^{*}(\cdot ), {T}^{*} (\cdot )\right)$ be the associated unique optimal solutions of the optimal control problem (10) with initial condition (11) and objective functional (12) with fixed final time ${T}_{f}$ (13). Then there exists adjoint function ${\lambda }_{i}^{*}(\cdot ), i = 1, ... , 4$ satisfying the following canonical equations:

$$\frac{{d\lambda_{1} }}{dt} = - \frac{{\partial {\mathcal{H}}}}{\partial S} = \left( {1 - {\mathfrak{u}}_{1} } \right)\beta A\left( {\lambda_{1} - \lambda_{2} } \right) + \mu \lambda_{1}$$

$$\frac{{d\lambda_{2} }}{dt} = - \frac{{\partial {\mathcal{H}}}}{\partial E} = \gamma \left( {\lambda_{2} - \lambda_{3} } \right) + \mu \lambda_{2}$$

$$\frac{{d\lambda_{3} }}{dt} = - \frac{{\partial {\mathcal{H}}}}{\partial A} = - A + \left( {1 - {\mathfrak{u}}_{1} } \right)\beta S\left( {\lambda_{1} - \lambda_{2} } \right) + {\mathfrak{u}}_{2} \delta \left( {\lambda_{3} - \lambda_{4} } \right) + \mu \lambda_{3}$$

$$\frac{{d\lambda_{4} }}{dt} = - \frac{{\partial {\mathcal{H}}}}{\partial T} = \mu \lambda_{4} + \alpha \left( {\lambda_{4} - \lambda_{1} } \right)$$

with transiversality conditions

20 $$\lambda_{i}^{*} \left( {T_{f} } \right) = 0,i = 1, 2, \ldots ,4$$

Moreover, the corresponding optimal controls ${\mathfrak{u}}_{1}^{*}\left(t\right)$ and ${\mathfrak{u}}_{2}^{*}\left(t\right)$ are given by

$${\mathfrak{u}}_{1}^{*} \left( t \right) = \max \left\{ {0, min\left\{ {\frac{{\beta SA\left( {\lambda_{2} - \lambda_{1} } \right)}}{{{\mathfrak{B}}_{1} }},1} \right\}} \right\}$$

21 $${\mathfrak{u}}_{2}^{*} \left( t \right) = \max \left\{ {0,min\left\{ {\frac{{\delta A\left( {\lambda_{3} - \lambda_{4} } \right)}}{{{\mathfrak{B}}_{2} }} ,1 - g} \right\}} \right\}$$

From the previous analysis, to get the optimal point, we have to solve the system

$$\frac{{dS^{*} }}{dt} = \mu N + \alpha T^{*} - \left( {1 - {\mathfrak{u}}_{1} } \right)\beta S^{*} A^{*} - \mu S^{*} ,$$

$$\frac{{dE^{*} }}{dt} = \left( {1 - {\mathfrak{u}}_{1} } \right)\beta S^{*} A^{*} - \left( {\mu + \gamma } \right)E^{*} ,$$

$$\frac{{dA^{*} }}{dt} = \gamma E^{*} - \left( {\mu + {\mathfrak{u}}_{2} \delta } \right)A^{*} ,$$

$$\frac{dT}{{dt}} = {\mathfrak{u}}_{2} \delta A^{*} - \left( {\mu + \alpha } \right)T^{*} ,$$

with the Hamiltonian $\mathcal{H} ={\mathfrak{w}}_{1}{A}^{*}+\frac{{\mathfrak{B}}_{1}}{2}{({\mathfrak{u}}_{1}^{*})}^{2}+\frac{{\mathfrak{B}}_{2}}{2}{({\mathfrak{u}}_{2}^{*})}^{2}+{\lambda }_{1}\left(\mu N+\alpha {T}^{*}-\left(1-{\mathfrak{u}}_{1}\right)\beta {S}^{*}{A}^{*}-\mu {S}^{*}\right)+{\lambda }_{2}\left(\left(1-{\mathfrak{u}}_{1}\right)\beta {S}^{*}{A}^{*}-\left(\mu +\gamma \right){E}^{*}\right)+{\lambda }_{3}\left(\gamma {E}^{*}-\left(\mu +{\mathfrak{u}}_{2}\delta \right){A}^{*}\right)+{\lambda }_{4}\left({\mathfrak{u}}_{2}\delta {A}^{*}-\left(\mu +\alpha \right){T}^{*}\right)$.

## Numerical analysis

### Numerical methods

In this study we have applied the numerical MATLAB ode45 programming code written by using the Runge–Kutta methods which are generally more powerful method for solving non-stiff ordinary differential equations.

### Numerical simulations for the deterministic model

In this section, we convey the numerical simulations to verify the theoretical results of our mathematical model (3). Particularly, some numerical justifications are considered to illustrate the theoretical analysis and results of the preceding sections. Here we assume the parameter values for numerical simulations that are not from real data, since there is the lack of mathematical modeling analysis literatures which have been done to study the dynamics of animosity towards mathematics. The initial values of the model (2) are positive, i.e.,$s\left(0\right)>0 ,e\left(0\right)\ge 0 ,v\left(0\right)\ge 0 ,z\left(0\right)\ge 0$ since the model represents human population. To understand the dynamics of students’ animosity towards mathematics, we need to assume parameter values and extremely analyze the model (3), and further explore how these parameters influence on the spread of animosity. For instance parameter describes the possibility that $s$(t) converts to be an $e(t)$ due to the presence of single $i$(t) per unit time. It is essentially determined by the rate of an $i$(t) spreading animosity.

#### Numerical simulation when the threshold quantity ${\mathcal{R}}_{0}$ less than unity

In this subsection let us consider the mathematical model (3) with the initial condition $\left({s}_{0}, {e}_{0}, {v}_{0}, {z}_{0}\right)=(0.74, 0.09, 0.07, 0.06, 0.04)$ and assume parameter values as $\beta =0.05,$ $\gamma =0.035,$ $\alpha =0.03,$ $\mu =0.02,$ $\delta =0.01$, and hence the basic reproduction number of model (2) is ${\mathcal{R}}_{0}=0.97$, Theorems 3.2 and 3.4 confirms that the animosity-free equilibrium point is locally and globally stable.

**Figure 2:**
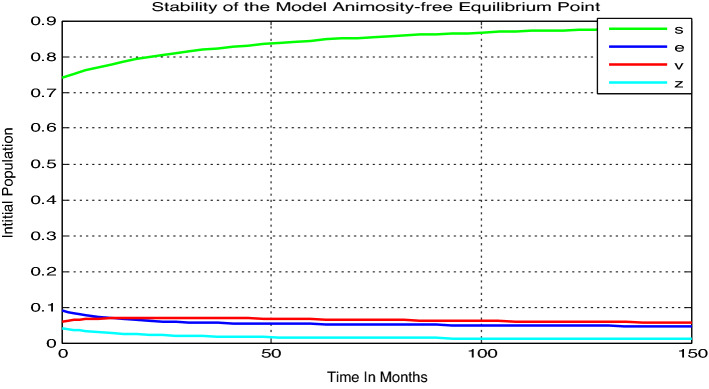
Local and global stability of the model animosity-free equilibrium point whenever ${\mathcal{R}}_{0}=0.97.$

Here from Fig. 2 we can justify that the model animosity-free equilibrium point is both locally and globally stable whenever ${\mathcal{R}}_{0}=0.97<1$, practically it means that animosity towards mathematics eradicate from the group of students in the near future (after 150 months) through the community.

#### Numerical simulation when the threshold quantity ${\mathcal{R}}_{0}$ greater than unity

In this sub-section let us consider the parameter values as $=0.5$ , $\gamma =0.04$, $\alpha =0.03$, $\mu =0.02$, $\delta =0.01$, then we have got the basic reproduction number of model (2) as ${\mathcal{R}}_{0}=11.1$, and we confirmed that the animosity-dominance equilibrium point is locally and globally stable.

**Figure 3:**
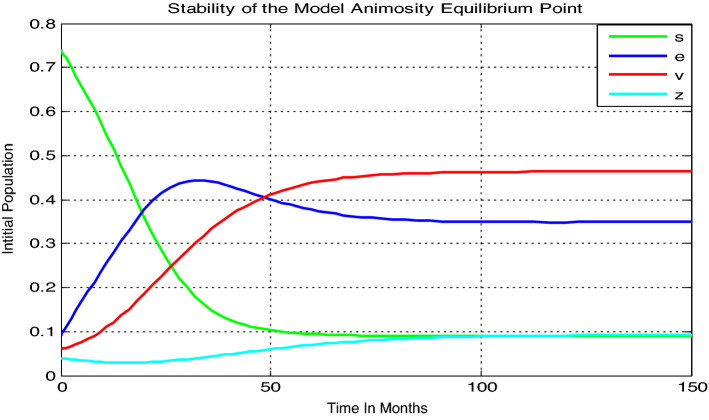
Local and global stability of the model animosity endemic equilibrium point whenever ${\mathcal{R}}_{0}=11.1.$

Here from Fig. 3 we can justify that the model animosity-dominance equilibrium point is both locally and globally stable whenever ${\mathcal{R}}_{0}=11.1>1$, practically it means that animosity of students towards mathematics exists uniformly after 150 months in the group of students throughout the community.

#### Effect of the transmission ${\varvec{\beta}}$ on the animosity-exposed students

**Figure 4:**
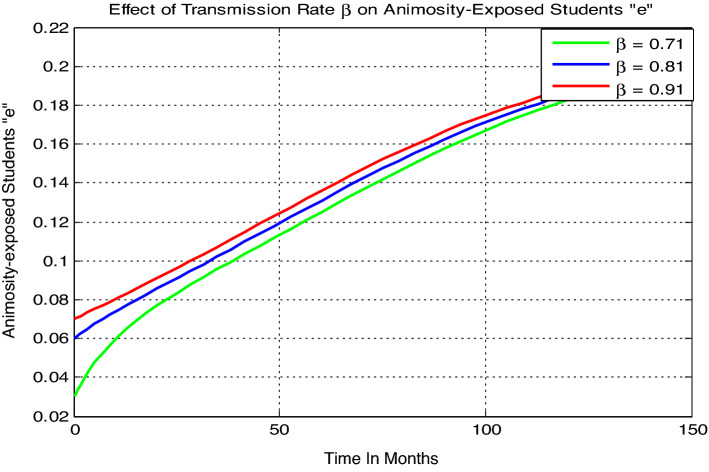
Effect of transmission rate $\beta$ on animosity-exposed students.

Here we perform numerical simulation of animosity-exposed students “e” with parameters values given by $\gamma =0.035$, $\alpha =0.03,\, \mu =0.02,\, \delta = 0.01$, and variable transmission rate $\beta$. Here from Fig. 4 we can justify that the animosity-exposed number of students “e” is going up whenever the contact rare or transmission rate $\beta$ increases.

#### Effect of ${\varvec{\gamma}}$ on the animosity-infected students

**Figure 5:**
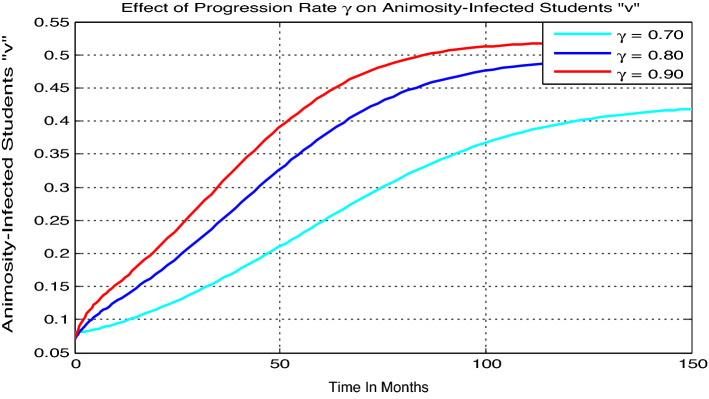
Effect of progression rate $\gamma$ on Animosity-infected students.

Here we simulate the model (2) animosity-infection with parameters values given by $\beta = 0.04$, $\gamma =0.035$, $\mu =0.02$, $\delta =0.01$, with variable value of $\gamma$. Figure 5 reflects that whenever the value of $\gamma$ increases the number of animosity-infected students “$v$” going up.

#### Effect of ${\varvec{\delta}}$ on the animosity-infected students

**Figure 6:**
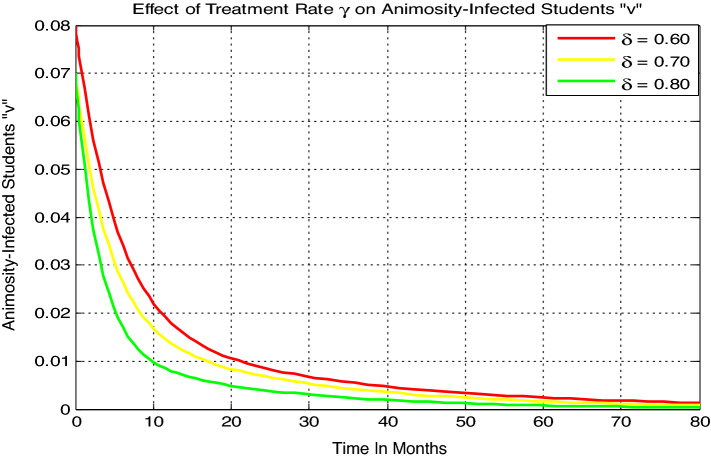
Effect of treatment rate $\delta$ on animosity-infected students.

Here we simulate the model (2) animosity-infection with parameters values given by $\beta =0.04$, $\gamma = 0.035,\, \mu =0.02,\, \delta =0.01$, with variable value of $\delta.$ Figure 6 reflects that whenever the value of treatment rate $\delta$ increases then the number of animosity-infected students “$v$” going down. For numerical simulations performed and shown by Fig. 2 up to Fig. 6 the parameter values we have taken have very crucial effect to verified the analytical results examined in Sect. "Qualitative investigation of the deterministic model" and to understand the students’ animosity towards mathematics in universities under consideration also to show how to minimize and possibly eradicate the animosity infection from the students population under the study area.

### Optimal control model simulations

In this section we perform numerical simulations of the optimal control problem for a period of 250 to 300 months and illustrate analytical results using MATLAB ode45 software where the positive valued constant, ${\mathfrak{w}}_{1}$ is the weight constant which represents the weight which balance offs the animosity infected students and the positive constants ${\mathfrak{B}}_{1}$, and ${\mathfrak{B}}_{2}$ represent the weight constants for the efforts on preventing animosity infection and improved animosity treatment respectively. The values assigned to the weight constants are ${\mathfrak{w}}_{1}=1$, ${\mathfrak{B}}_{1}=100$, and ${\mathfrak{B}}_{1}=100$ as given in^3^.

#### Optimal control simulation when both controls are applied

**Figure 7:**
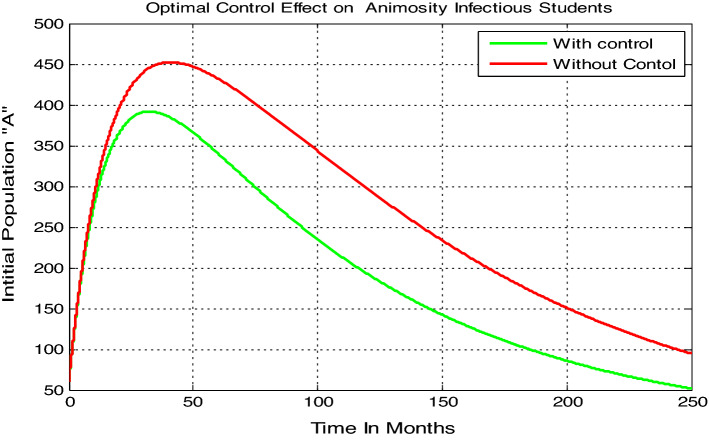
Variation in animosity infected students with and without both the controls.

Here we carried out numerical simulation on the model (1) animosity-infectious class ($A$) with parameters values given by $\beta =0.04$, $\gamma =0.035$, $\mu =0.02$, $\delta =0.01$ by applying both controls (i.e. ${u}_{1}\ne 0$ and ${u}_{2}\ne 0$), it results in significant difference in the number of infections under the application of controls which is shown in Fig. 7. We note that for the class of animosity infected students there is huge decrease in the number of infections when both controls are applied, and the number nears to zero after 250 months.

#### Optimal control simulation when exclusively one of the controls is applied

**Figure 8:**
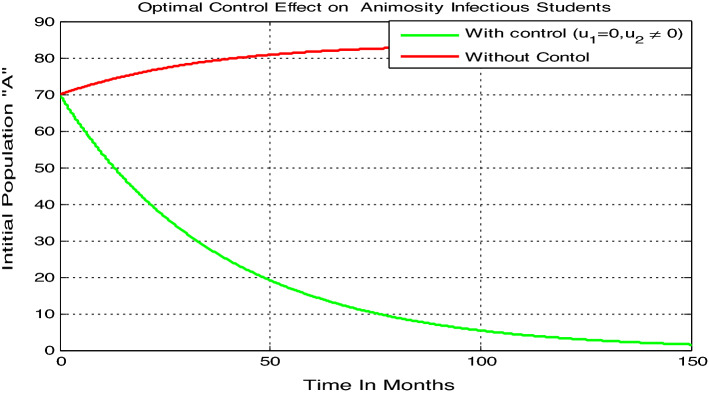
Variation in animosity infected students with treatment (${u}_{1}=0$ , ${u}_{2}\ne 0$) control only.

Here we carried out numerical simulation on the model (1) animosity-infectious class ($A$) with parameters values given by $\beta =0.04$, $\gamma =0.035$, $\mu =0.02$, and $\delta =0.01$, by applying only treatment control (i.e. ${u}_{1}=0$ and ${u}_{2}\ne 0$ ), it results in significant difference in the number of infections under the application of treatment only control which is shown in Fig. 8. We note that for the class of animosity infected students there is a decrease in the number of infections when treatment only control is applied, and the number nears to zero after 150 months and whenever without also treatment control i.e., when we do not apply both controls measures the animosity infectious class is increasing and becomes constant after 150 months.

**Figure 9:**
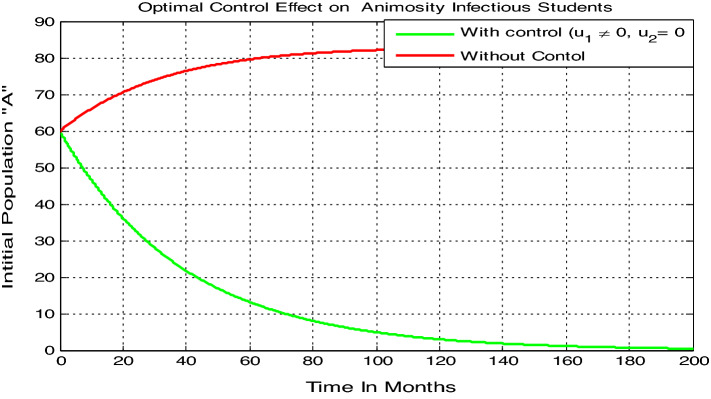
Variation in animosity infected students with prevention (${u}_{1}\ne 0$ , ${u}_{2}=0$) control only.

Here we carried out numerical simulation on the model (1) animosity-infectious class ($A$) with parameters values given by $\beta =0.04$, $\gamma = 0.035$, $\mu = 0.02$, $\delta = 0.01$, by applying only prevention control (i.e. ${u}_{1}\ne 0$ and ${u}_{2}=0$), it results in significant difference in the number of infections under the application of prevention only control which is shown in Fig. 9. We note that for the class of animosity infected students there is a decrease in the number of infections when prevention only control is applied, and the number nears to zero after 200 months and whenever without also prevention control the animosity infectious class is increasing and becomes constant after 200 months. Finally, parameter values used to simulate Figs. 7, 8, and 9 have a fundamental impact to show how to control the students’ animosity towards mathematics using suitable control measures applied in the model formulation.

## Conclusion

In this study we performed a detailed study on the deterministic with some stochastic touch mathematical model on the students’ animosity towards mathematics with optimal control approach comprising of four mutually exclusive compartments. The study began with a detailed analysis covering equilibriums, basic reproduction number, and stability analysis of the disease-free and endemic equilibriums. From the analysis on students animosity towards mathematics model we have proved that the disease-free equilibrium and the endemic equilibrium are locally asymptotically stable when the basic reproduction number (${\mathcal{R}}_{0}$) is less than unity and greater than unity respectively.

The theoretical results are then verified by optimal control analysis and numerical simulations respectively and results shown us the following:

- The model solutions converging to the animosity infection-free equilibrium point whenever ${\mathcal{R}}_{0}=0.97<1,$ biologically the animosity infection will be eradicate in the near future.
- The model solutions converging to the animosity-dominance equilibrium point around 250 months whenever ${\mathcal{R}}_{0}=11.1>1,$ biologically the animosity infection will be spreading in students.
- The animosity-exposed number of students “e” is going up whenever the contact rare or transmission rate $\beta$ increases, whenever the progression rate $\gamma$ increases the number of animosity-infected students “$v$” going up, and whenever the value of treatment rate $\delta$ increases then the number of animosity-infected students “$v$” going down.

Optimal control analysis performed by including two control parameters, one is the prevention mechanism $({u}_{1}(t))$ and the other is treatment of animosity infection mechanisms such as psychological treatment, tutorial methods $({u}_{2}(t)).$ A significant difference in the number of infections under the application of controls was shown in the results discussions. The effect of these controls individually was also performed and when we applied both controls together, the reduction in the number of infected population was quite large. Hence, this analysis focused on the need of improved prevention mechanisms and treatment to minimize and possibly to eradicate the animosity from students population. Hence, the results from the study imply acceleration in the animosity spread if proper prevention and treatment are not implemented. On a concluding note, the study further suggests that in the times of animosity spreading prevention and treatment should not be neglected. Although some literatures have acknowledged university students animosity towards mathematics, no study has attempted to explore the idea with mathematical modelling approach. This study attempts to contribute to the educational research by formulating and analyzing a mathematical modelling approach with optimal control approach on animosity towards mathematics which allows for deep understanding and a more comprehensive idea on the thematic area and it is distinguished from previous studies by using mathematical modelling approach as infectious disease to examine animosity towards mathematics.

## Data Availability

All data generated or analyzed during this study are included in this published article.
